# Endothelin-1-Induced Microvascular ROS and Contractility in Angiotensin-II-Infused Mice Depend on COX and TP Receptors

**DOI:** 10.3390/antiox8060193

**Published:** 2019-06-22

**Authors:** Christopher S. Wilcox, Cheng Wang, Dan Wang

**Affiliations:** Division of Nephrology and Hypertension, Department of Medicine, Georgetown University, Washington, DC 20007, USA; wilcoxch@georgetown.edu (C.S.W.); wangch2@mail.sysu.edu.cn (C.W.)

**Keywords:** endothelin 1 (ET-1), reactive oxygen species (ROS), cyclooxygenase (COX), thromboxane prostanoid receptors (TPRs), micro-resistant vessels, superoxide dismutase (SOD), nicotinamide adenine dinucleotide phosphate (NADPH) oxidase, tempol

## Abstract

(1) Background: Angiotensin II (Ang II) and endothelin 1 (ET-1) generate reactive oxygen species (ROS) that can activate cyclooxygenase (COX). However, thromboxane prostanoid receptors (TPRs) are required to increase systemic markers of ROS during Ang II infusion in mice. We hypothesized that COX and TPRs are upstream requirements for the generation of vascular ROS by ET-1. (2) Methods: ET-1-induced vascular contractions and ROS were assessed in mesenteric arterioles from wild type (+/+) and knockout (−/−) of COX1 or TPR mice infused with Ang II (400 ng/kg/min × 14 days) or a vehicle. (3) Results: Ang II infusion appeared to increase microvascular protein expression of endothelin type A receptors (ETARs), TPRs, and COX1 and 2 in COX1 and TPR +/+ mice but not in −/− mice. Ang II infusion increased ET-1-induced vascular contractions and ROS, which were prevented by a blockade of COX1 and 2 in TPR −/− mice. ET-1 increased the activity of aortic nicotinamide adenine dinucleotide phosphate (NADPH) oxidase and decreased superoxide dismutase (SOD) 1, 2, and 3 in Ang-II-infused mice, which were prevented by a blockade of TPRs. (4) Conclusion: Activation of vascular TPRs by COX products are required for ET-1 to increase vascular contractions and ROS generation from NADPH oxidase and reduce ROS metabolism by SOD. These effects require an increase in these systems by prior infusion of Ang II.

## 1. Introduction

Angiotensin II (Ang II) contributes to much of the vasculopathy and organ dysfunction in hypertension [1], diabetes mellitus [2], chronic kidney disease (CKD) [3], and aging [4]. All of these conditions are accompanied by systemic oxidative stress and can be ameliorated or prevented in animal models by the administration of effective antioxidant drugs, such as tempol [5,6,7,8].

Reactive oxygen species (ROS) contribute to hypertension and vasoconstriction with Ang II or endothelin 1 (ET-1) [9,10,11,12,13,14]. ROS can activate cyclooxygenase (COX) 1 [15,16,17] and 2 [3,15] and promote signaling via thromboxane prostanoid receptors (TPRs) [5,15] and thereby can enhance vasoconstriction [18,19]. These data demonstrate that ROS can be upstream activators of signaling by COX and TPRs, but the interactions are complex and not completely understood [20]. Indeed, an infusion of Ang II into mice generates oxidative stress, as indexed by increased 8-isoprostane F_2α_, renal vasoconstriction, and hypertension that are reduced or absent in TPR −/− mice [9,21]. This demonstrates that generation of ROS during Ang II infusion can be a downstream product of an activated COX/TPR pathway. However, this pathway has not previously been investigated in the vasculature. Ang II can generate ET-1. Therefore, we tested the hypothesis that ET-1 enhances ROS generation and the contractility of microvessels from Ang-II-infused mice by products of arachidonic acid generated by COX 1 and 2 that activate TPRs. COX1 and TPR wild type (+/+) and littermate knockout (−/−) mice were used to test the COX1 and TPR pathways directly. The effects of COX2 were assessed by a pharmacological blockade of COX2 in COX1 −/− mice to obviate any confounding effects from a nonselective COX blockade.

Ang II infusion generates ROS from nicotinamide adenine dinucleotide phosphate (NADPH) oxidase or from mitochondria and can decrease superoxide (O_2_^·−^) metabolism by decreasing superoxide dismutases (SODs) [14,22]. Therefore, we tested the activities of vascular NADPH oxidase and SODs to evaluate the origin of TPR-dependent changes in vascular ROS.

These experiments have a clinical impact since selective TPR antagonists are available and, in the absence of effective antioxidants, might provide a novel strategy to diminish vascular oxidative stress and its attendant morbidity.

## 2. Materials and Methods

### 2.1. Mice

This study used male 57Bl/6 mice (Charles river, Frederick, MD, USA) and TPR [9] and COX1 [23] gene-deleted (−/−) and littermate wild type (+/+) mice—all aged 4–5 months. The TPR mice were on a C57Bl/6J background [24] and the COX1 mice were on a Bl6/129 mixed background (Jackson Lab; Bar Harbor, ME 04609, USA). The TPR mice were generously provided by Thomas M Coffman (Division of Nephrology, Duke University, Durham, NC, USA) [24]. Both strains were backcrossed for more than nine generations and were inbred and genotyped in our laboratory. The mice were housed in a quiet room at 25 °C with a 12:12 h light-dark cycle and were provided with free access to food and water until the day of the study. All mouse experiments were performed with the approval of the Georgetown University Animal Care and Use Committee and in accordance with the guidelines of the Association for Assessment and Accreditation of Laboratory Animal Care—International (AAALAC) and the NIH Office of Laboratory Animal Welfare (OLAW) (The animal welfare assurance number is A3282-01).

### 2.2. Animal Model and Angiotensin II Infusion

**Figure 1:**
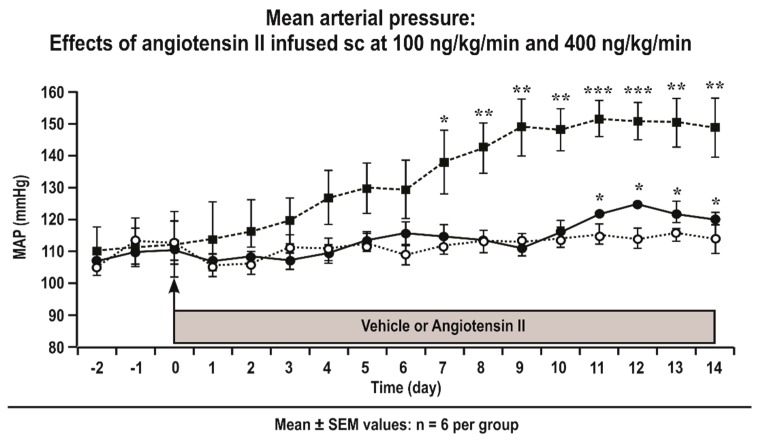
Angiotensin II infusion at 400 ng·kg^−1^·min^−1^ subcutaneously continuously administration (sc) is a slow pressor model, whereas angiotensin II infusion at 100 ng·kg^−1^·min^−1^ sc is a sub-threshold model. The daily average 24 h mean arterial pressure (MAP) was measured telemetrically in groups of eight conscious C57Bl/6 mice infused subcutaneously with angiotensin II at 400 ng·kg^−1^·min^−1^ (solid boxes and dashed lines) or 100 ng·kg^−1^·min^−1^ (solid circles and continuous lines) and sham-infused mice (open circles and dotted lines). Significance of change from sham: *, *P* < 0.05; **, *P* < 0.01; ***, *P* < 0.005.

Mice were anesthetized with 2% isoflurane in O_2_ for insertion of osmotic minipumps. Ang II (Peninsula Laboratory, San Carlos, CA, USA) was infused by osmotic minipumps (model 1002; Alza, Palo Alto, CA, USA) for 12–14 days [10] at 400 ng·kg^−1^·min^−1^ (Ang II 400) to provide a slow pressor infusion [10]. Some additional mice were infused with angiotensin II at 100 ng·kg^−1^·min^−1^ (Ang II 100) for 12–14 days to provide a threshold pressor infusion (Figure 1). Sham surgeries were performed in control mice (sham). The mean arterial pressure (MAP) of groups (*n* = 6) of conscious mice infused with Ang II (400 or 100 ng·kg^−1^·min^−1^ subcutaneously continuously administration (sc.) × 14 days) vs. sham mice were assessed telemetrically, as described [23]. Except where stated, studies were performed in mice infused with a slow pressor rate of Ang II (400 ng∙kg^−1^∙min^−1^ sc) or equivalent sham. After 12–14 days, the mice (*n* = 6–9 per group) were sacrificed and the mesenteric resistance arterioles were isolated and prepared as described [11].

### 2.3. Measurement of Urinary 8-Isoprostane F_2α_ and Thromboxane B_2_ (TxB_2_)

The other groups of mice (*n* = 6–7 per group) were housed in mouse metabolic cages (Nalgene Nunc International, Rochester, NY, USA) and urine was collected for 24 h, as described [5,22,25]. The 8-isoprostane F_2α_ and TxB_2_ in urine were purified, extracted, assayed (Enzo Life Science Inc. Farmingdale, NY, USA), and individual recoveries were assessed as described and validated [25,26]. The values were normalized with creatinine (Exocell, Philadelphia, PA, USA).

### 2.4. Protein Expression from Mesenteric Resistance Arterioles

The dilutions and sources of antibodies used were: COX1—1:1000 dilution (Cell signaling Technology, Danvers, MA, USA), COX2—1:1000 dilution (Cell signaling Technology, Danvers, MA, USA), TPR—1:1500 dilution (Thermo Scientific, Rockford, IL, USA), and endothelin type A receptors (ETARs)—1:1000 dilution (Thermo Scientific, Rockford, IL, USA). The protein levels were undertaken as described [27]. All the antibodies were diluted in double-deionized water.

### 2.5. Contractility and ROS Generation of Mesenteric Resistance Arterioles

Arterioles (mean luminal diameter 145 ± 6 μm and length circa 2 mm) were separated from the superior mesenteric bed and mounted on a myograph [11]. The vascular media and luminal areas were measured as described [28,29]. Concentration–response curves relative to a standard vascular contraction with 10^−7^ mol·L^−1^ norepinephrine plus 30 mmol·L^−1^ KCl (NAK) were obtained to phenylephrine (10^−8^ to 10^−5^ mol·L^−1^), U-46,619 (10^−9^ to 10^−5^ mol·L^−1^), and ET-1 (10^−10^ to 10^−7^ mol·L^−1^) and compared to a vehicle.

To evaluate the roles of ROS in the response to ET-1 (10^−7^ mol·L^−1^), vessels were incubated with tempol (10^−4^ mol·L^−1^) for 20 min [5]. To evaluate the roles of COX1 plus 2 or thromboxane A_2_ synthase (TxA_2_S), vessels were incubated with SC-560 (10^−6^ mol·L^−1^; SC, inhibitor of COX1; Sigma, St. Louis, MO, USA) plus paracoxib (10^−5^ mol·L^−1^; Para, inhibitor of COX2; Sigma, St. Louis, MO, USA) or OKY-046NA (10^−5^ mol·L^−1^; OKY, inhibitor of TxA_2_ synthase) for 30 min. These are the maximum effective concentrations [27]. COX1 +/+ and −/− and TPR +/+ and −/− mouse vessels were also used to evaluate the roles of COX1 and TPRs. ROS production with ET-1 (10^−^^7^ mol·L^−1^) was determined in vessels loaded with dihydroethidium (DHE), as described [27]. Fluorescence was quantitated by a PTI RatioMaster (Photon Technology International, London, ON, Canada) [27].

### 2.6. Measurement of Nicotinamide Adenine Dinucleotide Phosphate (NADPH) Oxidase and Superoxide Dismutase (SOD) Activities in Mouse Aortas

C57Bl/6 mice were infused with Ang II (400 or 100 ng·kg^−1^·min^−1^ sc × 14 days) or sham-operated and sacrificed. Fresh aortas were cut into separate pieces and incubated for 1 h with a vehicle or SQ-29,548 (10^−5^ M) to block the TPRs, followed by 4 h of ET-1 (10^−7^ mol·L^−1^) or vehicle. NADPH oxidase activity was assessed as described [22], and superoxide dismutase (SOD) activity was assessed using kits (STA-340, Cell Biolabs, Inc.). In order to measure SOD1, SOD2, and SOD3, the aortas were homogenized, and the activities of the SOD isoforms were assessed by SOD activity kits (Enzo Life Sciences, Cat, ADI-900-157, Farmingdale, NY 11735, USA). For SOD1 activity, SOD2 was inactivated in the homogenate by adding 400 µL of ice-cold chloroform/ethanol (37.5/62.5 (*v/v*)) to 250 µL of aorta lysate, shaking for 30 s, and centrifuging at 2500× *g* for 10 min. The upper aqueous phase yielded SOD1 activity. For SOD2, cyanide (2 mmol·L^−1^) was added to the homogenate to inhibit SOD1 activity. For SOD3 activity, the extracellular matrix was prepared with all cells removed by centrifuging at 250× *g* for 10 min at 4 °C. The assay of the supernatant represented the SOD3 activity.

### 2.7. Chemicals and Solutions

Agents were purchased from Sigma (St. Louis, MI, USA) and dissolved in a physiologic salt solution (PSS).

### 2.8. Statistical Analysis

Data are presented as mean ± SEM. Cumulative dose–response experiments were analyzed by nonlinear regression (curve fit) and differences were assessed by two-way, repeated-measures ANOVA with interaction followed, if appropriate, with Bonferroni post hoc *t*-tests for multiple comparisons. All the statistical analyses were performed using GraphPad Prism 7.0 software (Graphpad software, San Diego, CA 92108, USA). A probability value <0.05 was considered statistically significant.

## 3. Results

### 3.1. Mean Arterial Pressure (MAP) of Conscious Mice Infused with Angiotensin II

Telemetrically measured mean daily MAP increased significantly above levels of sham mice from Day 7 in C57Bl/6 mice infused with Ang II at 400 ng·kg^−1^·min^−1^ (Figure 1). In contrast, the MAP did not increase significantly above the levels of sham mice in mice infused with Ang II at 100 ng·kg^−1^·min^−1^ sc which is thus a sub-threshold dose (Figure 1).

### 3.2. Basal Values and Urinary Biomarkers in TPR +/+ and −/− and COX1 +/+ and −/− Mice

A slow pressor infusion of Ang II (400 ng·kg^−1^·min^−1^ × 12–14 days) increased the MAP of TPR +/+ and COX +/+ mice, but this was prevented in COX 1 and TPR −/− mice [9] (Table 1 and Table 2). The body, heart, and aorta weights were not different. The mesenteric resistance arteriolar media, lumen areas were increased by Ang II, which were the same as the urinary excretions of 8-isoprostane F_2α_ and TxB_2_, but these effects of Ang II were prevented in TPR −/− and COX1 −/− mice.

### 3.3. Protein Expression in Mesenteric Resistance Arterioles

**Figure 2:**
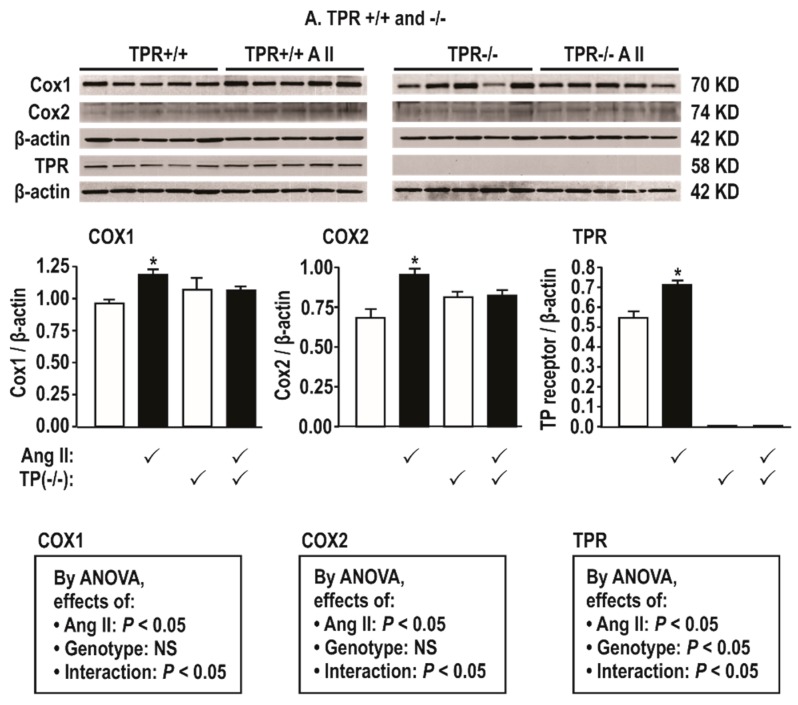
Angiotensin II infusion at 400 ng·kg ^−1^ min^−1^ × 14 days increases mesenteric resistance arteriolar protein expression for cyclooxygenase (COX) 1 and 2 and thromboxane prostanoid receptors (TPRs) in TPR wild type but not knockout mice. Mean ± SEM values (*n* = 5 per group). Compared to without angiotensin II infusion: *, *P* < 0.05.

**Figure 3:**
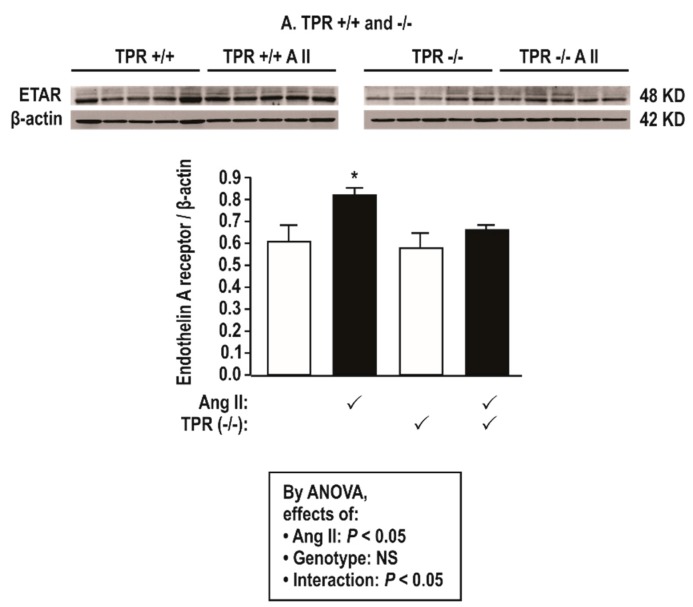
Angiotensin II infusion at 400 ng·kg^−1^ min^−1^ × 14 days increases mesenteric resistance arteriolar protein expression for endothelin type A receptors (ETARs) in thromboxane prostanoid receptor (TPR) wild type but not in knockout mice. Mean ± SEM values (*n* = 5 per group). Compared to without angiotensin II infusion: *, *P* < 0.05.

Ang II infusion (400 ng·kg^−1^·min^−1^) increased the mesenteric resistance arteriolar protein expression modestly for COX1 and COX2, TPR (Figure 2), and ETAR (Figure 3), but these changes were prevented in TPR −/− mouse vessels.

### 3.4. Contractile Responses of Mesenteric Resistance Arterioles to Phenylephrine (PE), U-46,619, and Endothelin 1 (ET-1) 

**Figure 4:**
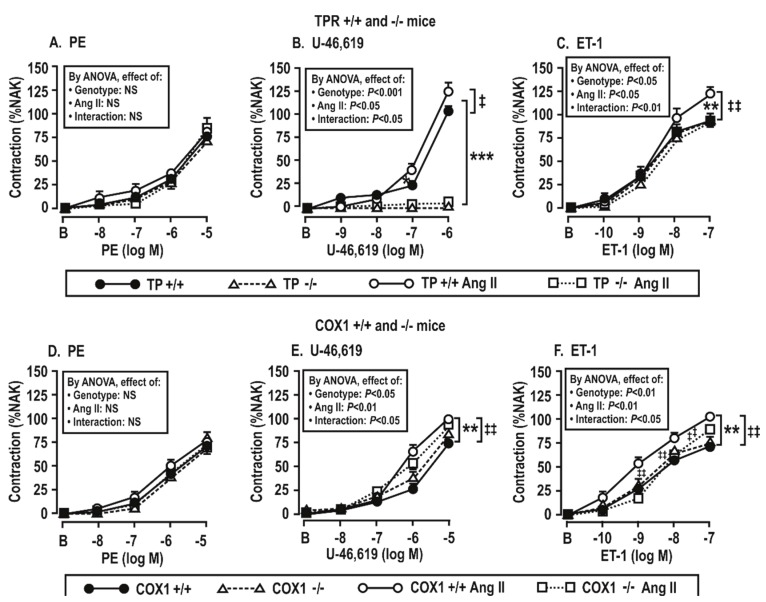
TPRs and COX1 are required for enhanced vascular contractions to U-46,619 (**B**,**E**) and endothelin 1 (**C**,**F**) but not to phenylephrine (PE, **A**,**D**) in mesenteric resistance arterioles from mice infused with angiotensin II at 400 ng·kg^−1^·min^−1^ × 14 days. Mean ± SEM values (*n* = 6 per group). For vascular contractions in thromboxane prostanoid receptor (TPR) wild type (+/+) and knockout (−/−) mouse vessels (upper panels) and COX 1 +/+ and −/− mouse vessels (lower panels). B, before; %NAK, compared to a standard vascular contraction with norepinephrine and high KCl. Compared to without angiotensin II infusion: *, *P* < 0.05; **, *P* < 0.01, ***, *P* < 0.005. Compared to +/+ mice: ‡‡, *P* < 0.01; NS: *P* > 0.05.

Vascular contractile responses to phenylephrine were not changed by the infusion of Ang II (Figure 4A,D), but vascular contractions to U-46,619 and ET-1 were increased significantly (*P* < 0.05) by the Ang II infusion in TPR +/+ or COX1 +/+ mouse vessels. However, these changes were reduced or prevented in TPR −/− and COX1 −/− mouse vessels (Figure 4B,C,E,F). As expected, vessels from TPR −/− mice had no contractions to U-46,619. 

### 3.5. Contractile Responses of Mesenteric Resistance Arterioles to Endothelin 1: Effect of Angiotensin II Infusion and of the Blockade of ROS, COX, and TxA_2_ Synthase in COX1 +/+ and −/− and TPR +/+ and −/− Mice 

**Figure 5:**
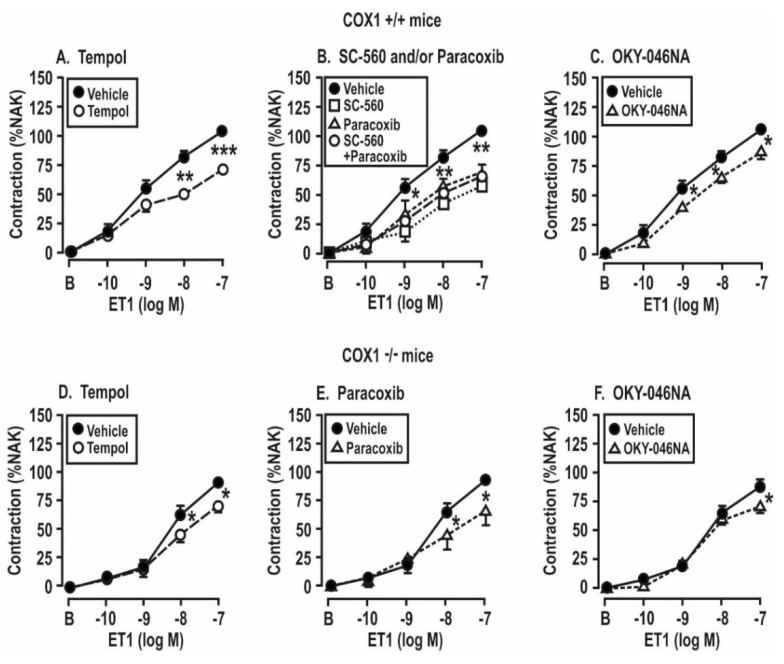
Contractions to endothelin 1 in mesenteric resistance arterioles from mice with angiotensin II infusion at 400 ng·kg^−1^·min^−1^ sc × 14 days are reduced by the metabolism of reactive oxygen species (**A**,**D**) or by the blockade of cyclooxygenase 1 (**B**,**E**) or thromboxane synthase (**C**,**F**) in vessels from COX1 +/+ mice, but the effects are reduced in vessels from COX1 −/− mice. Mean ± SEM values (*n* = 6 per group) for the vascular contractions to endothelin 1. B, before; %NAK compared to standard vascular contraction with norepinephrine and high KCl. Comparing groups: *, *P* < 0.05; **, *P* < 0.01; ***, *P* < 0.005. The control data are reproduced from Figure 4.

**Figure 6:**
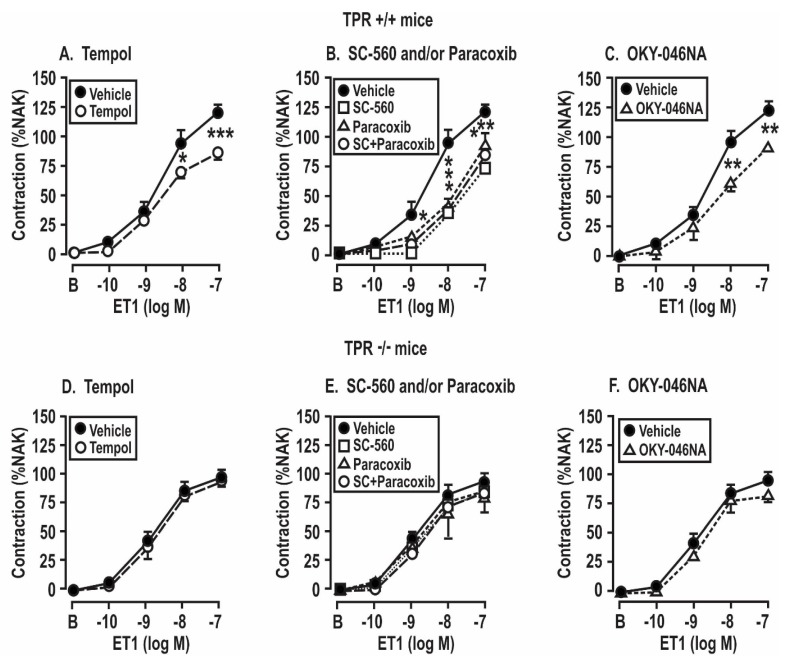
Contractions to endothelin 1 in mesenteric resistance arterioles from mice infused with angiotensin II at 400 ng·kg^−1^·min^−1^ × 14 days are reduced by the metabolism of reactive oxygen species (**A**,**D**) or by the blockade of cyclooxygenase (COX) 1 and 2 (**B**,**E**) or thromboxane synthase (TxA_2_S, **C**,**E**) but the effects are lost in vessels from thromboxane prostanoid receptor (TPR) −/− mice. Mean ± SEM values (*n* = 6 per group) for the vascular contractions to endothelin 1 in wild type (+/+) and thromboxane prostanoid receptor knockout (−/−) mice. B, before; % NAK, percent of the response to a standard vascular contraction with norepinephrine and high KCl solution. Comparing groups: *, *P* < 0.05; **, *P* < 0.01; ***, *P* < 0.005. The control data are reproduced from Figure 4.

These experiments were undertaken to assess the roles of angiotensin II infusion for 14 days and the products of COX 1 or 2 or TxA_2_ synthase in the enhanced contractions to ET-1 shown in COX1 +/+ vs. −/− and TPR +/+ vs. −/− mice in Figure 5 and Table 3. These enhanced contractions and the effects of the drugs (tempol, SC560, paracoxib, and OKY-046NA) added to the bath to block the ROS, COX1 or 2 and TxA_2_S were apparent only in the mesenteric arterioles from mice infused with angiotensin II for 14 days and only in wild type of TPRs and COX1 mice. Therefore, the studies presented in Figure 5; Figure 6 were undertaken in arterioles from Ang-II-infused mice. We had shown that contractions to PE were not dependent on TPRs or ROS [27]. Moreover, Figure 4 demonstrates that contractions to PE were not dependent on the angiotensin II infusion, COX1, or TPRs. Therefore, contractions to PE were not studied further in Figure 5 and Figure 6.

Contractions to ET-1 were not affected by the COX1 or TPR genotypes or by the addition of drugs to the bath in mesenteric arterioles from mice infused for 14 days with a vehicle (Table 3). However, in the arterioles from mice infused with angiotensin II for 14 days, contractions to ET-1 were significantly reduced (*P* < 0.05) in vessels from TPR +/+ and COX1 +/+ mice by the inactivation of ROS with tempol, the blockade of COX1 with SC-560 or COX1 and 2 with SC-560 and paracoxib, and the blockade of thromboxane A_2_ synthase with OKY-046NA (Figure 5A–C). The effects of these drugs on ET-1-induced contractions were reduced more modestly in vessels from COX1 −/− mice (Figure 5D–F). Contractions to ET-1 in vessels from TPR +/+ mice were also significantly reduced by tempol, SC-560 and/or paracoxib, and OKY-046NA (Figure 6A–C), whereas the effects of these drugs on ET-1-induced contractions were prevented in TPR −/− mouse vessels (Figure 6D–F).

### 3.6. Generation of Reactive Oxygen Species (ROS) with Endothelin 1 in Mesenteric Resistance Arterioles

**Figure 7:**
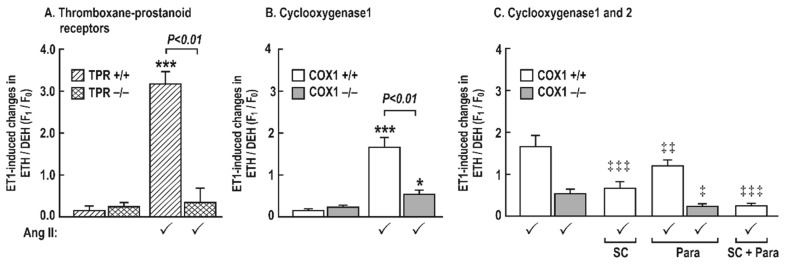
The changes in the generation of reactive oxygen species after a 5 min incubation with endothelin 1 (10^−7^ mol∙L^−1^) in mesenteric resistance arterioles from mice with angiotensin II infusion at 400 ng·kg^−1^·min^−1^ sc × 14 days is reduced in vessels from cyclooxygenase 1 −/− mice or by blockade of cyclooxygenase (COX) 1 (**B**) or 2 (**C**) and is prevented in vessels from thromboxane prostanoid receptor (TPR) −/− mice or by combined blockade of COX1 and 2 (**A**). Mean ± SEM values (*n* = 8 per group) for ROS generation (ethidium (ETH)—dihydroethidium (DEH) fluorescence ratio) was increased in the in response to endothelin 1 (10^−7^ mol·L^−1^) in vessels incubated for 5 min with a vehicle, SC-560 (SC; 10^−6^ mol·L^−1^), and/or paracoxib (Para; 10^−5^ mol·L^−1^). Effect of angiotensin II infusion: * *P* < 0.05; ***, *P* < 0.005. Effects of SC-560 or paracoxib; ‡, *P* < 0.05; ‡‡, *P* < 0.01; ‡‡‡, *P* < 0.005.

There was little ROS generated in the mesenteric resistance arterioles incubated with ET-1 in vehicle-infused mice (Figure 7). However, in vessels from mice infused with angiotensin II (400 ng∙kg^−1^∙min^−1^ sc × 14 days), ET-1 increased ROS generation greatly in TPR +/+ mice (3.18 ± 0.30 vs. 0.13 ± 0.13 Δunits, *P* < 0.001) and COX1 +/+ (1.68 ± 0.17 vs. 0.1 ± 0.006 Δunits, P < 0.001) mice, but this increase in ROS with ET-1 was reduced in vessels from COX1 −/− mice (0.1 ± 0.1 vs. 0.4 ± 0.1 Δunits, *P* < 0.05) and was fully prevented in vessels from TPR −/− mice (0.3 ± 0.2 vs. 0.2 ± 0.1, NS) (Figure 7). Moreover, the ROS generation in vessels from COX1 +/+ mice after a combined blockade of COX1 (SC-560; 10^−6^ mol·L^−1^) and 2 (paracoxib; 10^−5^ mol·L^−1^) was completely blocked (0.1 ± 0.1 vs. 0.2 ± 0.1, Δunits, NS) as in vessels from COX1 −/− mice after a bath addition of paracoxib (0.2 ± 0.1 vs. 0.2 ± 0.2, Δunits, NS). ROS generation with ET-1 in vessels from COX1 +/+ mice was reduced, but not prevented, after a blockade of COX1 (0.7 ± 0.1 vs. 1.6 ± 0.3, Δunits, *P* < 0.01) or COX2 (1.1 ± 0.1 vs. 1.6 ± 0.3, Δunits, *P* < 0.05) (Figure 7).

### 3.7. Activities of Nicotinamide Adenine Dinucleotide Phosphate (NADPH) Oxidase and Superoxide Dismutase (SOD) Isoforms in Aortas Stimulated by Endothelin 1 and Incubated with a Thromboxane Prostanoid Receptor Blocker or a Vehicle in Mice Infused with Angiotensin II at a Slow Pressor or Sub-Threshold Rate

**Figure 8:**
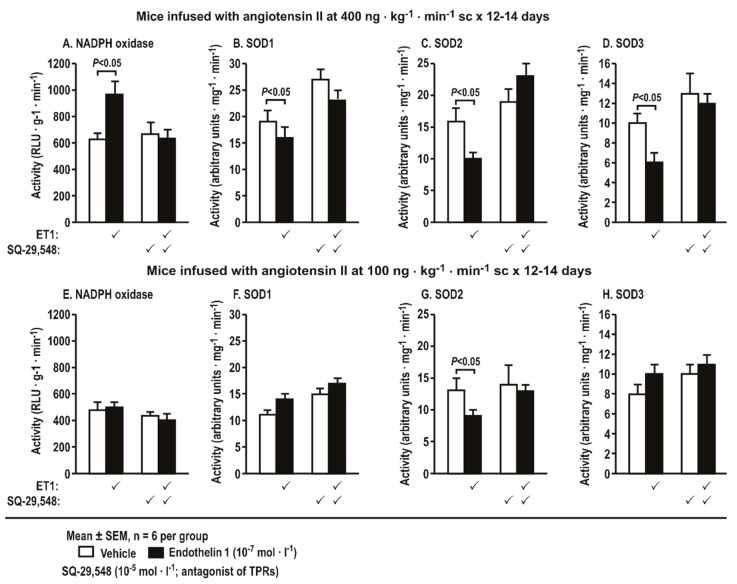
Incubation of aortas with endothelin 1 (10^−7^ mol·L^−1^ × 4 h) from mice with angiotensin II infusion at a slow pressor rate of 400 ng·kg^−1^·min^−1^ sc × 14 days increases the activity of nicotinamide adenine dinucleotide phosphate (NADPH) oxidase (**A**) and decreases the activity of superoxide dismutase (SOD) isoforms (**B**,**F**: SOD1; **D**,**H**: SOD3), whereas only SOD2 activity (**C**,**G**) is decreased by ET-1 after infusion with angiotensin II at a sub-pressor rate of 100 ng·kg^−1^·min^−1^. These effects are prevented by a blockade of thromboxane prostanoid receptors with SQ-29,548 (10^−5^ mol·L^−1^). Mean ± SEM values (*n* = 6 per group).

Incubation of aortas with ET-1 (10^−7^ mol·L^−1^ × 4 h) from mice infused with Ang II at a slow pressor rate (400 ng·kg^−1^·min^−1^ sc × 14 days) increased the activity of NADPH oxidase but decreased the activities of SOD1, 2, and 3. All of these changes were blocked by SQ-29,548 (Figure 8A). Incubation of aortas with ET-1 (10^−7^ mol·L^−1^ × 4 h) from mice infused with Ang II at a sub-threshold rate (100 ng·kg^−1^·min^−1^ SC-560 × 12–14 days) decreased the activity of SOD2. This was also blocked by SQ-29,548, but, otherwise, the sub-pressor infusion of Ang II did not change the activities of NADPH oxidase or the other SOD isoforms (Figure 8B).

These studies extend our prior reports that a prolonged slow pressor infusion of Ang II into mice enhances vascular contractions to ET-1 and U-46,619, but not PE, and that these depend on ROS and TPRs [5,11]. The main new findings are that a slow pressor infusion of Ang II modestly increases the mesenteric resistance arteriolar protein expressions of COX1 and 2, TPRs, and ETARs, however, these changes are reduced or prevented in vessels from TPR −/− mice. Likewise, hypertension, microvascular remodeling, increased systemic oxidative stress, and TxA_2_ production during slow pressor infusions of Ang II are prevented in TPR −/− mice. TPR knockout also prevents, and COX1 knockout moderates, the enhanced vascular contractions to U-46,619 and ET-1, however, these effects are confined to vessels from Ang-II-infused mice. Likewise, the generation of ROS with ET-1 is confined to the vessels from mice infused with Ang II at a slow pressor rate and depends on COX1 and 2 and TPRs. Finally, a TPR blockade prevents the enhanced activity of NADPH oxidase and reduced aortic activities of SOD1, 2, and 3 in aortas stimulated with ET-1 from mice infused with Ang II at a slow pressor rate and prevents the reduced activity of SOD2 with ET-1 in aortas from mice infused with Ang II at a sub-threshold rate.

## 4. Discussion

### 4.1. ROS are Downstream from the Activation of COX or TPRs in Mice Infused for Two Weeks with Ang II

These studies extend the prior reports that ROS are upstream from COX and TPRs [18,19] and demonstrate that ROS stimulated by ET-1 are also downstream from COX and TPRs. On the one hand, ET-1 can be released from blood vessels [18] and the kidney [19] by ROS generated by Ang II, yet ET-1 itself can generate ROS in blood vessels by the activation of NADPH oxidase and by the inhibition of SOD1, 2, and 3 in our study or by xanthine oxidase and mitochondrial cytochrome oxidase [30]. The generation of ROS by ET-1 was minimal or absent in mesenteric resistance arterioles unless harvested from Ang-II-infused mice where it became robust. This may be explained by prior findings that the Ang II infusion increased the expression of p22^phox^ [11], p47^phox^ [12], p67^phox^ [31], and NOX-2 and increased the activity of NADPH oxidase while reducing the activity of SOD isoforms in the kidney [32] and blood vessels [13] in this study. Thus, prolonged exposure to Ang II primes the blood vessels to generate superoxide (O_2_•^−^) from NADPH oxidase and other sources upon stimulation with ET-1. The present studies show further that Ang II infusion reduces O_2_•^−^ metabolism by SOD1, 2, and 3 and discloses a previously unrecognized absolute requirement for COX1 and/or 2 and TPRs to mediate the enhanced vascular contractions and ROS generation with ET-1. Thus, activated COX1 and/or COX2 and TPR signaling pathways are required for ROS generation with ET-1 during prolonged slow pressor infusions of Ang II.

COX1 and 2, TPR, and ETAR protein expression in mesenteric resistance arterioles are increased by two weeks of Ang II infusion and enhanced further by a positive feedback effect of thromboxane generation—COX is both a site for ROS generation [33] and a target for activation by ROS [3]. Ang II modestly increased the expression of COX2 in blood vessels in this study, and, although the expression of COX1 is constitutive, Ang II also increased its expression. Interestingly, the increased expression of both COX1 and COX2 by Ang II were dependent on TPRs. Since COX1 and 2 products activate TPRs, this suggests that ROS generated by the activation of TPRs during Ang II infusion can upregulate COX1 and 2 expressions to generate more ligands for TPR activation that itself upregulates COX1 and 2 in a positive feedback mode. This extends prior reports that TPR activation with U-46,619 increases the vascular conversion of radio-labeled arachidonate to prostaglandins and thromboxane [34]. Ang II failed to increase TxB_2_ excretion with Ang II in TPR −/− mice, suggesting a failure to activate COX and/or TxA_2_ synthase. Moreover, TxA_2_ generation in rats infused with U-46,619 enhances renal vasoconstriction [35]. Thus, there can be a positive feedback of TxA_2_ generation during the activation of TPRs whereby TxA_2_ activates COX1 and 2 and TPRs that themselves activate COX1 and 2 to sustain the increased ROS generation with Ang II.

### 4.2. Both COX1 and 2 Participate in ET-1-Induced ROS Generation in Mesenteric Resistance Arterioles from Ang-II-Infused Mice

The differentiation between the effects of COX1 and 2 has been hampered by cross-reactivity of the blocking drugs [36]. Therefore, we used COX1 −/− mice to define signaling via COX1, and COX1 −/− mice with administered with a COX2 antagonist (paracoxib) to define signaling via COX2 where there could be no cross-reactivity via COX1. Vascular contractions and ROS generation with ET-1 in vessels from Ang-II-infused mice were blunted after the pharmacological inhibition of COX1 or COX2 in COX1 −/− mice but were prevented in COX1 −/− mouse vessels by a COX2 blockade. Thus ET-1 increases ROS in the vessels from Ang-II-infused mice via both vascular COX1 and 2 isoforms.

### 4.3. Sources of ET-1 Stimulated ROS in Blood Vessels of Ang-II-Infused Mice

Ang II infusion increased the contraction of mesenteric resistance arterioles to U-46,619. This may be secondary to the generation of ROS by Ang II since H_2_O_2_ stabilizes the TPRs in cell membranes and increase TPR reactivity [37]. The upregulation of ETAR expression of mesenteric resistance arterioles with Ang II also depends on TPRs, likely reflecting TPR signaling via mitogen-activated protein kinases that enhance ETAR expression in vascular smooth muscle cells [38]. ET-1 increases vascular ROS in mice infused with Ang II at pressor rates by the activation of NADPH oxidase and by the reduction of all three SOD isoforms. Other studies have reported that ET-1 can activate mitochondrial ROS production [30]. The effect of ET-1 to stimulate ROS from mitochondria may be explained in part by the finding that SOD2 activity (mitochondrial SOD) was reduced by ET-1 in the aortas of mice infused with Ang II at a slow pressor rate. The blockade of TPRs in the mouse model of oxidative stress attenuates oxidative stress and improves SOD2 activity by reducing tyrosine nitration by peroxynitrite [39]. This may have contributed to the reduced SOD2 activity in aortas from mice infused with Ang II.

### 4.4. COX1 and 2 and TPRs are Absolute Requirements for Microvascular ROS Generation with ET-1 in Ang-II-Infused Mice

The most striking finding in this study was the absolute requirements for COX1 plus 2 and TPRs for ROS generation with ET-1 in microvessels from mice infused with Ang II. It demonstrates that prior exposure to Ang II is required to increase the expression of the machinery for ET-1-induced ROS generation (ETAR, COX1 and 2, and TPRs), that ET-1 then can utilize these to generate TxA_2_ via COX1 or 2 and TxA_2_ synthase, and that TxA_2_ activates TPRs to increase ROS generation by increasing NADPH oxidase and reducing ROS metabolism by SOD.

## 5. Conclusions

The activation of vascular TPRs by COX products are required for ET-1 to increase vascular contractions and ROS generation from NADPH oxidase and to reduce ROS metabolism by SOD. These effects require an increase in these systems by prior infusion of Ang II. 

## 6. Clinical Perspectives

The finding in this study that TPRs mediate many vascular effects of prolonged Ang II exposure [40] could be assessed in clinical studies using available TPR antagonists [27,41] to determine whether they could be beneficial in protecting blood vessels and organs from oxidative damage and inflammation in states of increased Ang II [15,27,42].

## Figures and Tables

**Table 1:** Basal parameters in conscious mice for mean arterial pressure, body, heart, and aorta weight; mesenteric resistance arterioles (MRA) remodeling; and urinary biomarkers in thromboxane prostanoid receptor (TPR) +/+ and −/− mice infused with angiotensin II (Ang II) or vehicle.

Variable	Vehicle		Ang II Infusion		By ANOVA, Effect of		
	TPR +/+	TPR −/−	TPR +/+	TPR −/−	TPR Genotype	Ang II	Interaction
MAP (mmHg)	92 ± 3	94 ± 2	108 ± 4 *	97 ± 3	NS	*P* < 0.05	*P* < 0.05
Body weight (g)	27.5 ± 0.5	27.0 ± 0.7	26.5 ± 0.7	27.4 ± 0.8	NS	NS	NS
Heart weight/Bwt (g)	0.99 ± 0.05	1.09 ± 0.10	0.89 ± 0.08	0.95 ± 0.12	NS	NS	NS
Aorta (mg)	0.15 ± 0.01	0.16 ± 0.007	0.16 ± 0.005	0.15 ± 0.006	NS	NS	NS
MRA lumen (µm)	139 ± 12	135 ± 6	121 ± 8	134 ± 5	NS	NS	NS
MRA media (µm)	42 ± 5	43 ± 7	51 ± 5 *	48 ± 6	NS	*P* < 0.05	NS
MRA M/L ratio	0.30 ± 0.06	0.31 ± 0.09	0.42 ± 0.08 *	0.35 ± 0.07 †	NS	*P* < 0.05	*P* < 0.05
Urinary 8-Isoprostane F_2α_ (ng/mg creatinine)	1.43 ± 0.18	1.79 ± 0.11	2.23 ± 0.13 *	1.66 ± 0.07 †	NS	*P* < 0.05	*P* < 0.05
Urinary TxB_2_ (ng/mg creatinine)	1.18 ± 0.07	0.92 ± 0.12	1.80 ± 0.13 *	1.18 ± 0.0 †	*P* < 0.05	*P* < 0.05	NS

Mean ± SEM, *n* = 6/group. Mice were infused with a vehicle or angiotensin II (400 ng·kg^−1^·min^−1^ sc × 14 days). MAP, mean arterial pressure; MRA, mesenteric resistance arterioles; TxB_2_, thromboxane B_2_. Compared to the vehicle: *, *P* < 0.05. Compared to corresponding TPR +/+ mice: †, *P* < 0.05. NS: *P* > 0.05.

**Table 2:** Basal parameters in conscious mice for mean arterial pressure, body, heart, and aorta weights, mesenteric resistance vessel (MRA) remodeling, and urinary biomarkers in cyclooxygenase (COX) 1 +/+ and −/− mice infused with angiotensin II (Ang II) or vehicle.

Variable	Vehicle		Ang II Infusion		By ANOVA, Effect of		
	COX1 +/+	COX1 −/−	COX1 +/+	COX1 −/−	COX1 Genotype	Ang II	Interaction
MAP (mmHg)	88 ± 3	94 ± 3	124 ± 4 *	112 ± 3†	*P* < 0.05	*P* < 0.05	*P* < 0.05
Body weight (BW, g)	26.5 ± 0.6	28.0 ± 0.8	27.5 ± 0.6	29.4 ± 0.9	NS	NS	NS
Heart weight (g)	0.94 ± 0.05	0.98 ± 0.11	1.05 ± 0.07	0.99 ± 0.12	NS	NS	NS
Aorta (g)	0.15 ± 0.01	0.16 ± 0.007	0.16 ± 0.005	0.15 ± 0.006	NS	NS	NS
MRA lumen (µm)	142 ± 9	135 ± 5	123 ± 7	132 ± 5	NS	NS	NS
MRA media (µm)	43 ± 4	41 ± 5	55 ± 6 *	47 ± 5	NS	*P* < 0.05	NS
MRA M/L ratio	0.30 ± 0.07	0.31 ± 0.04	0.44 ± 0.05 *	0.36 ± 0.06	NS	*P* < 0.05	*P* < 0.05
Urinary 8-Isoprostane F_2α_ (ng·mg creatinine-1)	1.44 ± 0.10	1.45 ± 0.22	2.11 ± 0.19 *	1.79 ± 0.21†	NS	*P* < 0.05	*P* < 0.05
Urinary TxB_2_ (ng·mg creatinine-1)	1.0 ± 0.05	0.99 ± 0.06	1.32 ± 0.07 *	1.03 ± 0.06 †	NS	*P* < 0.05	NS

Mean ± SEM, *n* = 6/group. Mice were infused with vehicle or angiotensin II (400 ng·kg^−1^·min^−1^ sc × 12–14 days). MAP, mean arterial pressure; MRA, mesenteric resistance arteriole; TxB_2_, thromboxane B_2_. Compared to vehicle, *, *P* < 0.05. Compared to corresponding COX1 +/+ mice: †, *P* < 0.05, NS: *P* > 0.05.

**Table 3:** Contractions to endothelin 1 (ET-1) in mesenteric resistance arterioles (MRA) incubated with physiological salt solution (PSS) or with drugs that block ROS (tempol), cyclooxygenase 1 (SC-560), cyclooxygenase 2 (paracoxib), or thromboxane A_2_ synthase (OKY-046). Effects of infusion of a vehicle or angiotensin II at a slow pressor rate for two weeks and of thromboxane prostanoid receptors (TPRs) or cyclooxygenase 1 (COX1).

**Added to the Bath**	**Vehicle Infusion**		**Ang II Infusion**		**By ANOVA, Effect of**		
	TPR +/+	TPR −/−	TPR +/+	TPR −/−	TPR Genotype	Ang II Infusion	Interaction
PSS (%)	94 ± 6	96 ± 3	122 ± 5 *^,^^†^	93 ± 7 ^†^	NS	*P* < 0.05	*P* < 0.05
Tempol (%)	90 ± 5	91 ± 4	86 ± 5 ^#^	92 ± 5	NS	NS	NS
SC-560 (%)	89 ± 7	90 ± 5	74 ± 7 ^#^	86 ± 7	NS	NS	NS
Paracoxib (%)	91 ± 4	92 ± 3	94 ± 10 ^#^	82 ± 6	NS	NS	NS
SC + Paracoxib (%)	86 ± 7	88 ± 5	85 ± 7 ^#^	87 ± 2	NS	NS	NS
OKY-046NA (%)	88 ± 6	92 ± 4	90 ± 2 ^#^	94 ± 3	NS	NS	NS
**Added to the Bath**	**Vehicle Infusion**		**Ang II Infusion**		**By ANOVA, Effect of**		
	COX1 +/+	COX1 −/−	COX1 +/+	COX1 −/−	COX1 Genotype	Ang II Infusion	Interaction
PSS (%)	75 ± 3	76 ± 7	104 ± 3 *^,†^	88 ± 4 ^†^	NS	*P* < 0.05	*P* < 0.05
Tempol (%)	70 ± 4	71 ± 5	70 ± 3 ^#^	72 ± 6	NS	NS	NS
SC-560 (%)	69 ± 5		59 ± 9 ^#^				
Paracoxib (%)	72 ± 6	70 ± 4	76 ± 6 ^#^	67 ± 9	NS	NS	NS
SC + Paracoxib (%)	68 ± 5		64 ± 4 ^#^				
OKY-046NA (%)	72 ± 6	73 ± 6	86 ± 6 *^,#^	76 ± 6	NS	*P* < 0.05	NS

Mean ± SEM, *n* = 6/group for contractions, relative to a standard contraction with norepinephrine and potassium (NAK; see Figure 4, Figure 5 and Figure 6) with a maximum concentration of endothelin 1 (10^−7^ mol·L^−1^) in mesenteric resistance arterioles incubated for 30 min with PSS or with tempol (10^−4^ mol·L^−1^), SC-560 (10^−6^ mol·L^−1^), paracoxib (10^−5^ mol·L^−1^), or OKY-046NA (10^−5^ mol·L^−1^). Vessels were from mice infused for 14 days with a vehicle or angiotensin II (400 ng·kg^−1^·min^−1^ sc). Compared to the vehicle: *, *P* < 0.05. Compared to corresponding +/+ mice: ^†^, *P* < 0.05. Compared to corresponding PSS: ^#^, *P* < 0.05.
